# Multi-Branch Convolutional Neural Network for Automatic Sleep Stage Classification with Embedded Stage Refinement and Residual Attention Channel Fusion

**DOI:** 10.3390/s20226592

**Published:** 2020-11-18

**Authors:** Tianqi Zhu, Wei Luo, Feng Yu

**Affiliations:** College of Biomedical Engineering and Instrument Science, Zhejiang University, Hangzhou 310027, China; terryzhu@zju.edu.cn (T.Z.); willi4m@zju.edu.cn (W.L.)

**Keywords:** sleep stage scoring, neural network-based refinement, residual attention

## Abstract

Automatic sleep stage classification of multi-channel sleep signals can help clinicians efficiently evaluate an individual’s sleep quality and assist in diagnosing a possible sleep disorder. To obtain accurate sleep classification results, the processing flow of results from signal preprocessing and machine-learning-based classification is typically employed. These classification results are refined based on sleep transition rules. Neural networks—i.e., machine learning algorithms—are powerful at solving classification problems. Some methods apply them to the first two processes above; however, the refinement process continues to be based on traditional methods. In this study, the sleep stage refinement process was incorporated into the neural network model to form real end-to-end processing. In addition, for multi-channel signals, the multi-branch convolutional neural network was combined with a proposed residual attention method. This approach further improved the model classification accuracy. The proposed method was evaluated on the Sleep-EDF Expanded Database (Sleep-EDFx) and University College Dublin Sleep Apnea Database (UCDDB). It achieved respective accuracy rates of 85.7% and 79.4%. The results also showed that sleep stage refinement based on a neural network is more effective than the traditional refinement method. Moreover, the proposed residual attention method was determined to have a more robust channel–information fusion ability than the respective average and concatenation methods.

## 1. Introduction

**Figure 1:**
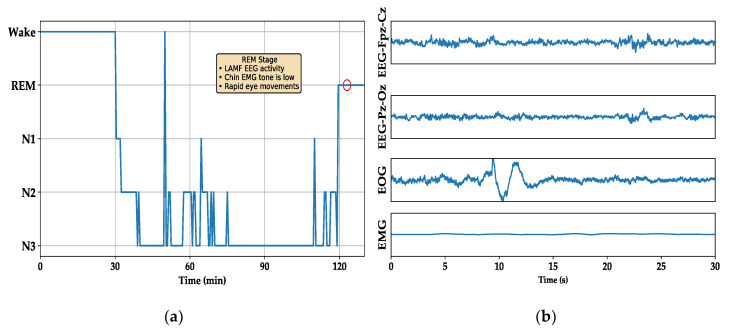
(**a**) Hypnogram of one sleep cycle. (**b**) Multi-channel physiological signal for the rapid eye movement (REM) stage corresponding to the red cycle in (**a**).

Sleep is an essential component of daily human activity. Obtaining adequate sleep is necessary for individuals to perform tasks when they are awake. Long-term lack of sleep can cause mental fatigue and impairment of decision-making and learning abilities. It can also cause migraines, Parkinson’s syndrome, and other diseases [1,2]. To effectively evaluate the human sleep state, researchers have established a scientific sleep evaluation and diagnosis system, such as the American Academy of Sleep Medicine Manual (AASM) [3]. In this guide, the sleep-related physiological signals collected by sensors are divided into wake, rapid eye movement, and nonrapid eye movement stages according to the signal characteristics. The nonrapid eye movement stage is further divided into N1, N2, and N3 stages according to the sleep depth. For example, the signal characteristics of the rapid eye movement (REM) stage (Figure 1b) are broadly described as low-amplitude mixed-frequency electroencephalography (EEG) activity, with minimal muscle activity and typical rapid eye movement. These activities are reflected in the signals collected by the corresponding sensors. According to the description of the characteristics of each stage in the AASM guide, an experienced medical technician must read each 30-s sleep epoch, one at a time, and determine the sleep stage to form a hypnogram, as shown in Figure 1a (only one cycle). This process is time-intensive and relies heavily on human resources. To alleviate this constraint, automated sleep staging based on modern signal processing technology can be used. This approach can significantly improve the efficiency of sleep disorder diagnosis.

**Figure 2:**
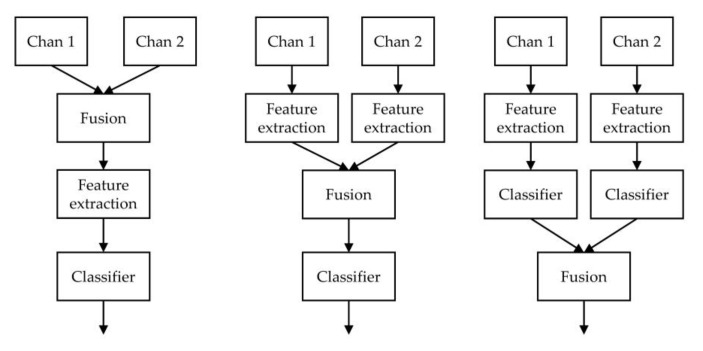
Diagrams of different channel fusion methods, which from left to right are data fusion, feature fusion, and decision fusion, respectively.

The automatic sleep stage classification process (Figure 2) can be divided into three steps—feature extraction, classification, and refinement—based on the transition rules of the stages. The first step is feature extraction. Before automatic feature extraction methods based on neural networks were developed, the features used for sleep staging were manually designed by researchers. Because brain activity changes in each sleep stage, the spectral energy density of the EEG signal in different frequency bands can effectively classify the signal. Other features used in sleep stage classification include spectral entropy [4], signal kurtosis [5], Hurst and fractal exponents [6,7], and others. With the development of deep learning methods in recent years [8,9,10], automatic feature extraction based on deep learning has become widely used in the biomedical field [11,12,13,14]. For example, Acharya et al. [15] used convolutional neural networks (CNNs) to classify Alzheimer’s disease lesions. Li et al. [16] used CNNs to classify EEG signals for motor image classification and achieved good results. For sleep staging classification, Supratak et al. [17] used a CNN to extract signal features and recurrent neural networks (RNNs) [18] to extract temporal features. Their technique achieved good performance on the Sleep-EDF dataset.

After the feature extraction step is completed, a classifier is selected in the second step to classify the features. Shi et al. [19] used the K-nearest neighbor (KNN) method, an unsupervised classifier based on joint sparse representation features. Other supervised methods for sleep staging include the support vector machine (SVM) [20], random forest [21], and neural network classifiers based on fully connected networks. Feature extraction by a CNN or RNN combined with a fully connected network classifier makes sleep staging classification an end-to-end process.

The last step is sleep stage refinement. As a cyclical process, the transition between adjacent sleep stages is not random, as shown in Figure 1a. Therefore, applying transition rules for refining the preliminary results can effectively improve the classification accuracy [22,23,24]. Because sleep continuity is one of the most important sleep transition rules, Liang et al. applied the rule to a smoothing method [22] to correct their preliminary results, such as by modifying (Wake, N1, Wake) to (Wake, Wake, Wake). Jiang et al. [23] employed a strategy based on the hidden Markov model (HMM) to refine the classification results and improve the classification performance. However, because the training process of end-to-end deep neural networks relies on the gradient backward propagation method, we cannot use these nondifferentiable traditional methods to build end-to-end models.

To score sleep stages, a single-channel signal or multi-channel signals are used. The selected type depends on the given application scenario. For example, owing to the limitation of collection equipment in wearable devices and household devices, it is more feasible to use single-channel signals in these scenarios. In clinical auxiliary diagnosis, methods based on multi-channel signals can obtain more reliable classification results [24,25,26,27,28]. Channel fusion is a crucial part of these methods. As shown in Figure 2, these methods can be divided into different fusion types—data-level, feature-level, and decision-level—according to the channel fusion stage. The fusion step that precedes feature extraction is called data fusion. As an example of data fusion, Phan et al. [28] proposed the conversion of EEG, electrooculography (EOG), and electromyography (EMG) signals into the frequency domain through a short-time Fourier transform. Then, a three-channel frequency image is applied as the input of the neural network model. 

Feature fusion methods often use a multi-branch feature extractor to separately extract features from different channels and then join the features [26] or superimpose them. They are then input into the classifier. Decision fusion involves the same feature extraction method as feature fusion; however, the channel fusion is placed after the classifier. These methods use voting or maximum posterior probability (MAP) [24] to determine the final category after each channel is independently classified. The channel fusion method often requires different choices according to the given task.

**Figure 3:**
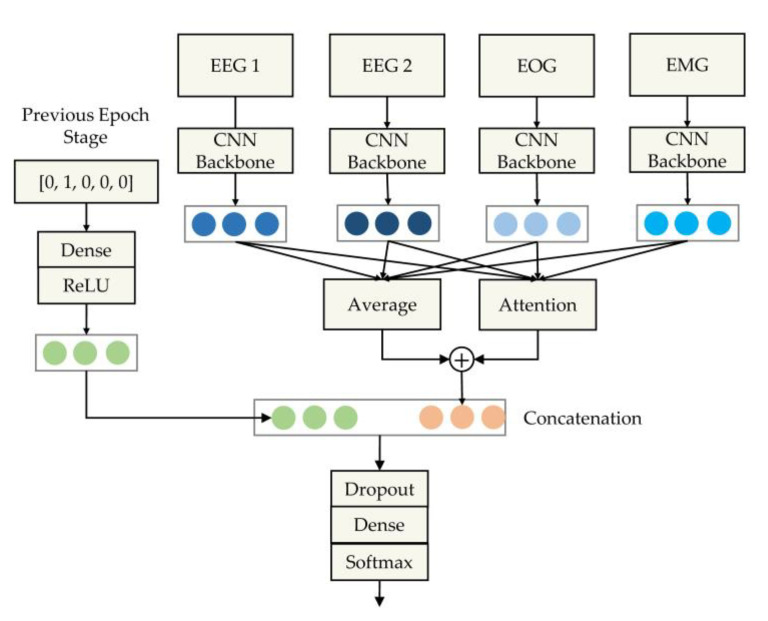
Schematic diagram of the proposed multi-branch convolution neural network combined with embedded stage refinement and residual attention-based channel fusion.

To achieve complete end-to-end processing and more effective use of multi-channel signal data, we propose a multi-branch convolutional neural network model with embedded stage refinement and a residual attention channel fusion method. The main contributions of the proposed approach are the following:

- Data from the previous sleep epoch stage are encoded through a neural network and embedded in the final classification process to correct the current stage. The proposed model thereby realizes end-to-end processing of feature extraction, classification, and transition refinement based on a deep neural network.
- The model uses multiple neural network branches (Figure 3) to extract the features of different channels. The model accounts for the importance of the channels in decision making. It thus employs a channel fusion method based on a self-attention mechanism [29] to dynamically adjust the decision weights of multiple channels.
- The model performance was evaluated on two public datasets. The model achieved better results than other state-of-the-art methods. At the same time, through these two datasets, we explained the performance differences of the model in stages.

## 2. Materials and Methods

### 2.1. Datasets

In this study, we employed two public datasets to evaluate the performance of the model: the Sleep-EDF Database Expanded (Sleep-EDFx) [30] and the University College Dublin Sleep Apnea Database (UCDDB) [31]. Both datasets are publicly available on PhysioNet [31] and contain multimodal and multi-channel data, including EEG, EOG, and chin EMG. The latest version of the Sleep-EDFx dataset contains two subsets—sleep cassette (SC) and sleep telemetry (ST)—which include a total of 104 individuals and 197 complete nights of polysomnographic sleep recordings. The data in the SC subset were collected from healthy individuals; the data in the ST subset were obtained from subjects who experience mild difficulty falling asleep. 

To facilitate a comparison of the proposed approach with existing methods, we employed the records of 39 nights of 20 individuals in the age range of 25 to 34 in the SC subset (the second night of subject 13 was missing). We adopted the same method in [17], which retained only portions of wake epochs. The Sleep-EDFx dataset contains two EEG channels, Fpz-Cz and Pz-Oz; one EOG channel; and one chin EMG channel. The sampling rate of the first three channels was 100 Hz, and the sampling rate of the EMG channel was 1 Hz. To maintain processing consistency, we upsampled the rate to 100 Hz. The UCDDB dataset contains complete night data of 25 individuals with sleep-disorder breathing collected at St Vincent’s University Hospital. The dataset includes two EEG channels, C3-A2 and C4-A1; two left and right EOG channels; and one chin EMG channel. The sampling rate of both EEG and EOG was 128 Hz, and the sampling rate of the EMG channel was 64 Hz. We upsampled the EEG channel to 128 Hz for the same reason as in Sleep-EDFx.

Here, we define as the “transition epoch” the epoch that occurs between the preceding epoch and the subsequent epoch. If the given epoch is not a transitional one, it is called a “nontransition epoch.” Table 1 shows the stage distribution of the two datasets and the distributions of the transition and nontransition epochs in each stage. It is observed in the table that the transition epoch ratio of stage N1 is significantly higher in both datasets than in the other stages. Furthermore, the UCDDB dataset has a higher transition epoch proportion than the Sleep-EDFx dataset.

Manual labeling of the two datasets was performed according to the R&K guideline [32], which defines six stages of sleep: Wake, N1, N2, N3, N4, and REM. Before their application as input to the model, the datasets were preprocessed in two simple steps. The first step was to merge N3 and N4 into a single stage, N3—also called the slow-wave stage—according to the AASM guidelines. The second step was to perform z-score normalization for each respective channel of each subject. That is, the mean and variance of each channel were respectively calculated and each channel was normalized.

### 2.2. Model Architecture

The overall architecture of the proposed model consists of four parts, as shown in Figure 3: a feature extraction module based on the convolutional neural network; a channel fusion module based on residual attention; an encoding module, which is used to embed the previous epoch stage into the model; and a classification module based on the fully connected layer.

In the feature extraction part of the proposed model, different branches of the CNN are used to extract the channel features of each channel signal. This means that the CNN does not share parameters between channels. The feature extraction module is composed of five convolutional blocks and a global average pooling layer. Each convolution block employs a convolution layer [10] to extract shift-invariant intermediate features. Each block also uses a batch normalization layer [33] to normalize the features to avoid model overfitting and to accelerate model convergence. Finally, the normalized features are input to the rectified linear unit layer (ReLU) [34] for nonlinear activation. The specific hyperparameters of the backbone are listed in Table 2.

We input the channel features obtained through the convolution backbone into the average module and the attention module. The outputs of these two modules are added to obtain the fused channel features. The full composition of these two modules is called the residual attention module. In this module, we mark the channel feature as $v_{i}$. The feature vector obtained by concatenating all channels is V. The channel weight $\alpha_{i}$ can be calculated by the following formula, where $W_{i}^{s}$, $W_{i}^{v}$, W, and b are learnable parameters:

(1) $$V=\mathrm{Concat}\left(\left[v_{1},{\;v}_{2},\cdots ,v_{C}\right]\right)$$

(2) $$s_{i}=W_{i}^{s}*\mathrm{ReLU}\left(W_{i}^{v}v_{i}+\mathrm{WV}+b\right)$$

(3) $$\alpha_{i}=\frac{\exp\left(s_{i}\right)}{\Sigma_{i=1}^{C}\exp\left(s_{i}\right)}$$

The weight of the channel may be saturated, which means that the model only focuses on one channel and completely ignores the other channels. To avoid the lack of information caused by this situation, we incorporate a threshold to the weight of each channel by adding the mean value of the multi-channel features to the output of the attention module. Therefore, the final fusion feature is

(4) $$V_{\mathrm{fusion}}=v_{\mathrm{avg}}+\Sigma_{i=1}^{C}\alpha_{i}v_{i}$$

Owing to the continuity of sleep and the hidden transition rules between adjacent sleep epochs, the current epoch stage can be corrected by using the stage information of the previous epoch. We use the truth value of the previous epoch during training. In addition, the probability vector of the model’s output at the previous epoch during inference is employed. To maintain consistency of the training and inference, we use one-hot encoding as input during training. Then, the model employs a single-layer fully connected layer to re-encode the stage into a vector of length 256 and concatenates it with the fused channel features. The connected vector passes through the classifier composed of the dropout layer [10], the fully connected layer, and the softmax layer. 

The fully connected layer is shown in equation (5). It is evident that the concatenation between the re-encoded stage vector ($v_{c}$) and the fused signal feature ($v_{\mathrm{fusion}}$) is equivalent to having an additive refinement for the probability vector ($P_{\mathrm{fusion}}$) corresponding to the fused feature vector. The dropout layer zeroes the neurons according to a certain probability to achieve regularization and avoid model overfitting. The softmax layer normalizes the stage probability vector for the loss calculation.

(5) $$P={\mathrm{Wh}}^{T}=\left[W_{c},W_{f}\right]{\left[v_{c},v_{\mathrm{fusion}}\right]}^{T}=W_{c}v_{c}+W_{f}v_{\mathrm{fusion}}=P_{c}+P_{\mathrm{fusion}}$$

### 2.3. Experiment Details

To evaluate the performance of the model on the entire dataset, we used the leave-one-subject-out method for testing. The experiment used one subject as the test set; the remaining subjects composed the training and validation sets. We repeated the experiment many times and then merged the results of each experiment on the test set to obtain the final result. For the Sleep-EDFx dataset, we set up 20 experiments, with each training set consisting of 18 subjects, and one individual composed the validation set. The remaining subjects composed the test set. The UCDDB dataset was used for a 25-fold experiment with 23 individuals as the training set and one subject as the validation set. We used cross-entropy as the loss function (Equation (6)), which $y_{i}$ is the one-hot label and $\hat{y_{i}}$ is predicted probability vector, and the Adam [35] optimizer to update the model parameters during training. The model and training parameters are summarized in Table 3.

(6) $$L=-\Sigma_{i\in \Omega }y_{i}\log\left(\hat{y_{i}}\right)$$

Owing to the small size of the training set, and to improve the generalization performance of the model on the test set during the inference process, we used an ensemble method. That method is based on multiple training epochs and infers the test set. The first step in the ensemble method is to use the model’s performance on the validation set as a reference to obtain the top 10% intermediate models during the training process, which included seven models in our experiment. Then, we used each intermediate model as an independent model to separately infer the test set. Finally, we used the maximum likelihood method to ensemble multiple results on the same test set and obtain the final results.

In this study, we employed accuracy, macro-average F1-score (MF1), and Cohen’s kappa coefficient to evaluate the overall performance of the model. Because MF1 and kappa provide different categories of the same importance, these two metrics could more reliably assess the actual performance of the model on the dataset with an imbalanced category distribution. In addition, MF1 was used as a verification indicator for selecting the training epoch during ensemble inference. Moreover, we calculated the precision, recall, and F1-score of each stage to evaluate the performance of the model in stages.

## 3. Results

### 3.1. Model Performance on Public Datasets

As evident in Table 4, the overall performance of the model on the two datasets has some gaps. The overall accuracy, MF1, and kappa of the Sleep-EDFx dataset are 85.8, 81.2, and 0.80, respectively, while the corresponding metrics of UCDDB are 79.4, 78.8, and 0.73, respectively. The performance of the proposed model is lower on UCDDB than on Sleep-EDFx. Specifically, the performance of stage N3 is comparable on the two datasets, the result of stage N2 is better than that of Sleep-EDFx on UCDDB. The performance in the other stages on UCDDB is significantly lower than on Sleep-EDFx. 

**Figure 4:**
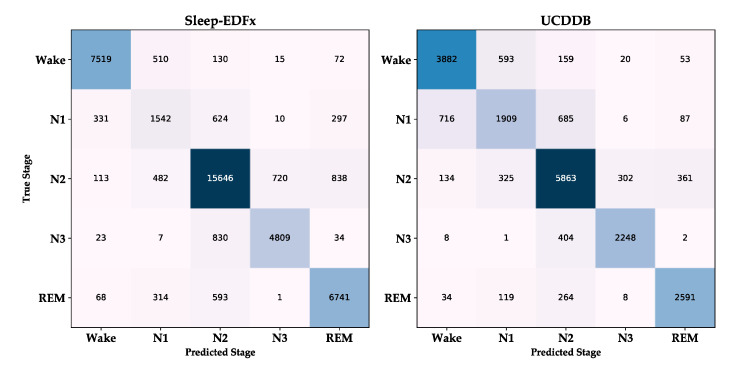
Classification confusion matrix of Sleep-EDFx and UCDDB.

The common point of the model performance on the two datasets is that stage N1 has the lowest accuracy, and the model performance has a clear gap in the other stages. The classification confusion matrix of the two datasets is shown in Figure 4. Their misclassification rules are the same. That is, the main misclassifications of Wake and REM are N1 and N2. N1 is more likely to be misclassified into stages other than N3. N2 has an even distribution of misclassifications, while N3 is almost only misclassified as N2.

Table 5 shows the performance comparison between the proposed model and existing models on the two datasets. The inclusion of a large number of wake stages will significantly improve the model performance. Moreover, subject-independent or nonindependent testing methods will also influence the model performance [17,25]. Therefore, we chose methods that use the same dataset. Furthermore, we employed a subject-independent testing method in the comparison of all models except that of [19], which only tested the nontransition epoch in the UCDDB dataset. It is observed in the table that our method is superior to the other methods in terms of accuracy, MF1, and kappa. Shi et al. [19] tested their model only on the nontransition window. It achieved an accuracy of 81%, while the accuracy of our model on the same data is 85.1%, and MF1 is 83.4%. The performance of our model is still better than that of Shi et al.

In addition, Zhang et al. [24] and Yuan et al. [40] used a multi-branch (MB) CNN-based multi-channel feature extraction method and a fully connected network-based classification method on the Sleep-EDFx and UCDDB datasets, respectively. Zhang et al.’s method uses decision-level channel fusion and expert rule-based refinement. Yuan’s method employs a multi-channel fusion method based on global information. Our method uses neural-network-based stage correction. Our channel fusion method based on residual attention is better than those methods.

### 3.2. Model Component Analysis

#### 3.2.1. Influence of Neural Network-Based Embedded Stage Refinement

To explore the effect of neural-network-based stage refinement on the model performance, we constructed a set of comparative experiments. The neural-network-based refinement module in the model was removed as the baseline model. The smoothing method based on expert rules [22] and the method based on the hidden Markov model (HMM) [23] were used to correct the stages. The rule-based refinement method set the matching rules and refinement rules. All sequences that met the matching rules were corrected according to the corresponding refinement rules. The specific rules are listed in Table 6. 

Refinement based on the hidden Markov model is divided into two steps. The first step was to use the ground truth stages of the validation set as the hidden state. The inference value of our model on the validation set was used as the observation value. Then, the state transition probability and emission probability of the HMM were obtained through a frequency-counting method. The second step was to maintain the HMM parameters as unchanged, and take the model’s inference result on the test set as the observation value, and obtain the hidden states of the HMM through the Viterbi [42] algorithm as the corrected results of the test set.

**Figure 5:**
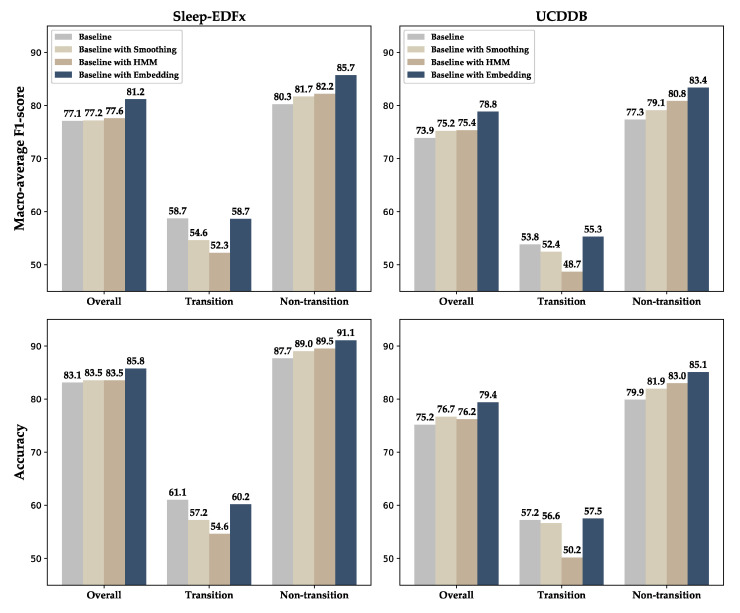
Performance comparison of different refinement methods on overall, transitional, and nontransitional epochs.

The comparison results are shown in Figure 5. The smoothing refinement and the HMM-based refinement improve the overall MF1 performance of the Sleep-EDFx, while the MF1 of the UCDDB dataset is slightly increased by 1.3% and 1.2%, respectively. It is apparent that these two methods improve the nontransition epoch performance by sacrificing the transition epoch performance. Moreover, the HMM-based correction method causes more degradation of the transition epochs performance, while engendering greater nontransition epoch improvement. In comparison, the refinement process based on the neural network improves the performance without causing the performance degradation of the transition epochs.

**Figure 6:**
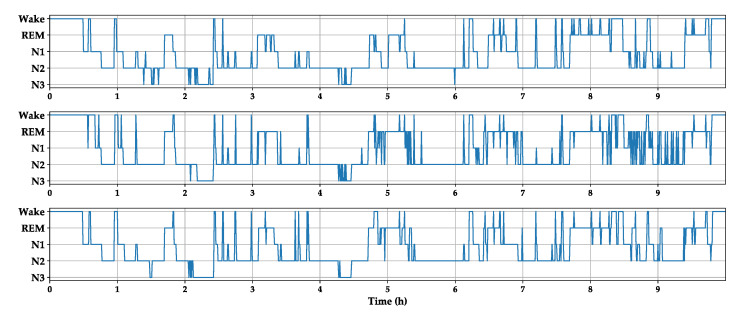
Hypnogram of subject 4 on the first night in the Sleep-EDFx. The graphs from top to bottom are derived from a human expert, baseline model, and baseline model with embedded stage refinement.

Figure 6 shows a hypnogram of subject 3 on the first night in the Sleep-EDFx dataset. From top to bottom is manual labeling by experts, the classification results based on the baseline model, and the results of the baseline neural network combined with embedded refinement. As shown in the figure, compared with the baseline model, the revised hypnogram is smoother and retains many critical transition processes that occur during sleep.

#### 3.2.2. Efficacy of Multi-Channel and Residual Attention

**Figure 7:**
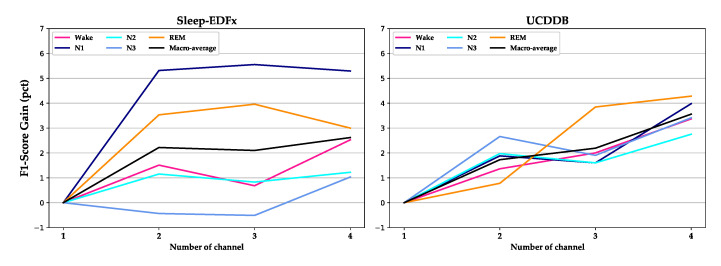
Performance gain for each stage by adding channels on Sleep-EDFx and UCDDB. The gain is computed by using the F1-score of each channel setting to subtract the F1-score of one channel.

In Figure 7, we show the performance gain obtained by multiple channels compared to a single channel. In the Sleep-EDFx dataset, the signals increase from one channel to four channels—Fpz-Cz, EOG, EMG, and Pz- Oz. The corresponding channels in the UCDDB dataset are C4-A1, EOG, EMG, and C3-A2. In the UCDDB dataset, each additional channel improves the overall performance, whereas on the Sleep-EDFx dataset, the performance decreases slightly after adding the EMG signal. This may have been due to the low sampling rate of the original EMG signal. Meanwhile, stage N1 and stage REM achieve greater performance improvement on the two datasets, which shows that the recognition process of N1 and REM staging is more dependent on multi-channel signal characteristics.

**Figure 8:**
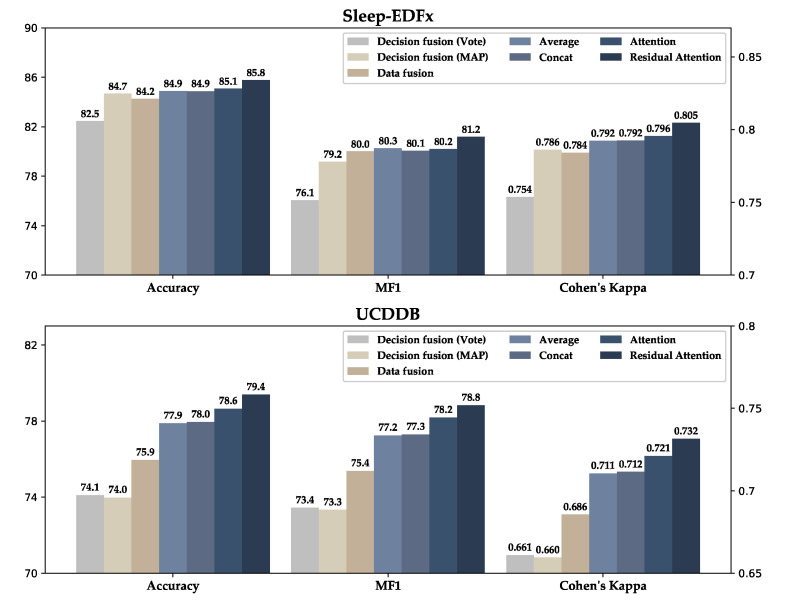
Performance comparison of different channel fusion methods.

Figure 8 shows the performance comparison of different channel fusion methods. In data fusion, the residual attention module is removed, and we use the same CNN backbone in which the four channels are concatenated with shape (3000, 4) as the model input. The decision fusion method trains an independent model for each channel. Then, the inference results of each model are fused by voting or the method of maximum posterior probability, as shown in Figure 2. 

In addition, we experimented with feature fusion methods based on the average, concatenation, and attention. It is observed from the results in Figure 8 that the performance of the method based on data fusion and decision fusion is lower than that of the method based on feature fusion. The performance of the model obtained by directly fusing the multi-channel features through average and concatenation is similar. After removing the average part of the proposed residual attention module, the model performance is slightly improved on the Sleep-EDFx dataset and it shows an accuracy increase of approximately 0.6% on the UCDDB dataset. The further application of the complete residual attention increases the classification accuracy of the model on the Sleep-EDFx dataset and UCDDB dataset by approximately 1% and 1.5%, respectively, and on MF1 by 1% and 1.5%, respectively.

**Figure 9:**
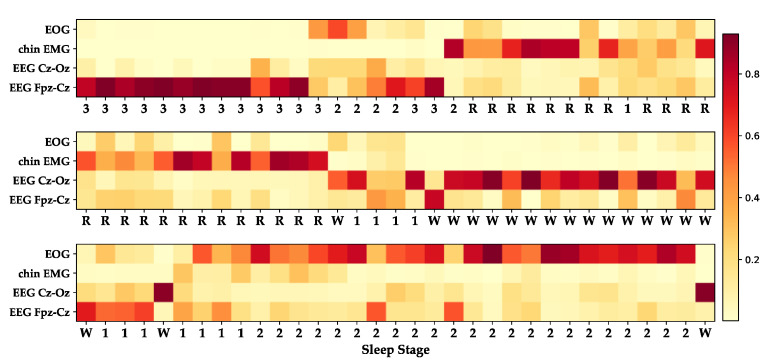
Attention weights for each channel in stage sequence, which W, 1, 2, 3, R refer to Wake, N1, N2, N3, and REM.

In Figure 9, we show the attention module’s weight response to different stages when the model uses the residual attention method. This stage sequence was obtained from the Sleep-EDFx dataset after subject 1 fell asleep on the first night at approximately 3.7 h to 4.5 h. It is observed on the heat map that the model mainly focuses on the Cz-Oz channel and the Fpz-Cz channel for the wake stage. For N1 and N3, the model confers greater weight to the Fpz-Cz channels. When identifying N2 and REM, the model mainly relies on EOG and EMG channels.

## 4. Discussion

On the basis of previous studies [17,24,36], we embedded the refinement stage into the neural network model and used the residual attention-based multi-channel signal fusion method to make the performance of our proposed model achieve the human expert level [43]. However, it should be noted that, similar to those of other methods, the performance of the proposed model for the different stages was inconsistent, and the accuracy of stage N1 was much lower than those of the other stages. In most previous studies, this was attributed to the underfitting caused by using fewer samples. However, in the UCDDB dataset, N1 had more samples than N3 and REM; however, its performance was far worse than in the other two stages. This shows that underfitting was only part of the cause rather than the main reason. From Table 1 and Figure 5, it is apparent that the actual cause of the poor performance of stage N1 was that the proportion of transition epochs in N1 was much higher than those in other stages. At the same time, the performance of the model on the transition epoch was much lower than that of the nontransition epoch. 

For example, on the Sleep-EDFx dataset, the proportion of the transition epoch in stage N1 is 55.4%, and the highest proportion in the other stages is 21.7% of stage N3. The MF1 of the model in the transition and nontransition epochs is 58.7% and 85.7%, respectively. Such a vast distribution and performance difference ultimately leads to the stage N1 performance being far lower than in the other stages. In addition, the UCDDB dataset is composed of individuals who have difficulty falling asleep. The proportion of the transition epoch is greater than that of the Sleep-EDFx dataset. Therefore, the performance of the model on UCDDB is lower than that on Sleep-EDFx.

Multi-channel data provides richer information than single-channel data. Nevertheless, owing to unreasonable channel fusion methods on occasion, more signal channels will not significantly improve the model [27]. It is also evident in Figure 8 that only using the self-attention module can only slightly improve the model. Figure 9 shows that the model can correctly learn the attention mode corresponding to the AASM guidelines. However, it is easier to have attention saturation on a specific channel. In other words, it completely ignores the information of other channels, which may affect the model performance. To solve this problem, we added the average module, which sets a threshold for the weight of each channel. The performance of the model was further improved by this method.

Based on the above experimental results and discussion, we found that the proposed approach still has some shortcomings. In future work, we will strive to address these limitations and improve our method. First, the refinement process of the model can significantly improve the performance of the nontransition epoch, but it cannot improve the performance of the transfer epoch, which also leads to a lower accuracy of stage N1. Therefore, we intend to develop models for the transition epoch, such as by enabling the model to identify the transition and nontransition epochs and by using different refinement modules to implement different correction strategies. Second, inter-individual differences also limit the generalization ability of the model across individuals. We will strive to use adversarial-learning-based methods to eliminate the differences between individuals and improve the model robustness.

## 5. Conclusions

In this paper, we proposed a multi-branch neural network model for automatic sleep stage classification. The model integrates feature extraction, feature classification, and stage refinement into a neural network model to form an end-to-end processing method. The model thereby significantly improves the performance of the nontransition epoch without lowering the accuracy of the transition epoch. In addition, the channel fusion method based on residual attention can further improve the performance of the model based on a naive fusion method (average, concatenation). On the Sleep-EDFx dataset composed of healthy individuals and the UCDDB dataset composed of patients with sleep-disorder breathing, the proposed model achieved better performance than existing models.

## Figures and Tables

**Table 1:** Details on the datasets used in this study.

Stage	Sleep-EDFx			UCDDB		
	Transition (%)	Nontransition	Total	Transition (%)	Nontransition	Total
**Wake**	844 (10.2)	7402	8246	1239 (26.3)	3468	4707
**N1**	1554 (55.4)	1250	2804	1349 (39.6)	2054	3403
**N2**	2873 (16.1)	14,926	17,799	1154 (16.5)	5831	6985
**N3**	1237 (21.7)	4466	5703	293 (11.0)	2370	2663
**REM**	761 (9.9)	6956	7717	258 (8.6)	2758	3016
**Total**	7268 (17.2)	35,001	42,269	4293 (20.7)	16,481	20,774

**Table 2:** Hyperparameters of convolution neural network backbone.

Layer Name	Kernel Size	Stride	Padding	Output Size	
				Sleep-EDFx	UCDDB
Input				[3000 × 1]	[3840 × 1]
conv_block_1	[1 × 5]	3	VALID	[999 × 64]	[1279 × 64]
conv_block_2	[1 × 5]	3	VALID	[332 × 64]	[425 × 64]
conv_block_3	[1 × 3]	2	VALID	[165 × 128]	[212 × 128]
conv_block_4	[1 × 3]	1	VALID	[163 × 128]	[210 × 128]
conv_block_5	[1 × 3]	1	VALID	[161 × 256]	[208 × 256]
global average pooling				[1 × 256]	

**Table 3:** Hyperparameters of the proposed neural network.

Model		Training	
Encoding size in embedded refinement	256	Batch size	64
Number of channels	4	Number of training epochs	70
Weights size in attention module	256	Loss function	Cross-entropy
Batch normalization (momentum and epsilon)	0.99, 0.001	Optimizer	Adam
Dropout rate	0.5	Learning rate	0.0001
		Clip value of the gradient	0.1

**Table 4:** Overall performance of the proposed model.

	W	N1	N2	N3	REM
**Sleep-EDFx**	**Accuracy: 85.8 Macro-Average F1-Score: 81.2 Cohen’s Kappa: 0.80**				
Precision	93.4	54.0	87.8	86.6	84.5
Recall	91.2	55.0	87.9	84.3	87.4
F1-score	92.3	54.5	87.8	85.4	85.9
**UCDDB**	**Accuracy: 79.4 Macro-Average F1-Score: 78.8 Cohen’s Kappa: 0.73**				
Precision	81.3	64.8	79.5	87.0	83.7
Recall	82.5	56.1	83.9	84.4	85.9
F1-score	81.9	60.1	81.7	85.7	84.8

**Table 5:** Performance of different methods on the Sleep-EDFx and UCDDB datasets.

Methods				Channels	Overall Performances		
Reference	Feature	Classifier	Refinement		Acc	MF1	Kappa
**Sleep-EDFx**							
Supratak et al. [17]	CNN & RNN	FC	-	1	82.0	76.9	0.76
Tsinalis et al. [36]	Handcraft	AE	-	1	78.9	73.7	-
Yu et al. [37]	ACNN	FC	-	1	82.8	77.8	-
Phan et al. [20]	ARNN	SVM	-	1	82.5	72.0	0.76
Mousavi et al. [38]	CNN & ARNN	FC	-	1	84.3	79.7	0.79
Zhang et al. [24]	MB-CNN	FC	Expert Rules	4	83.6	78.1	0.77
Proposed	MB-CNN	FC	FC	4	85.8	81.2	0.80
**UCDDB**							
Shi et al. [19]	Handcraft	RF	-	2	81.1	-	-
Martin et al. [39]	Handcraft	DBN	HMM	3	72.2	70.5	0.64
Yuan et al. [40]	MB-CNN	FC	-	4	74.2	68.2	-
Cen et al. [41]	CNN	FC	HMM	4	69.7	-	-
Proposed	MB-CNN	FC	FC	4	79.4	78.8	0.73

ACNN: attentional CNN, ARNN: attentional RNN, AE: auto encoder, DBN: deep brief nets.

**Table 6:** Refinement rules [22] (X represents any stage).

Rule No.	Matching Rules	Refinement Rules
1	Any REM epoch before the first appearance of N2	N1
2	Wake, REM, N2	Wake, N1, N2
3	N1, REM, N2	N1. N1, N2
4	N2, X, N2	N2, N2, N2
5	REM, X, REM	REM, REM, REM
